# Astroglial Plasticity Is Implicated in Hippocampal Remodelling in Adult Rats Exposed to Antenatal Dexamethasone

**DOI:** 10.1155/2015/694347

**Published:** 2015-08-04

**Authors:** Vishvesh H. Shende, Simon McArthur, Glenda E. Gillies, Jolanta Opacka-Juffry

**Affiliations:** ^1^Department of Life Sciences, University of Roehampton, London SW15 4JD, UK; ^2^Division of Experimental Medicine, Imperial College, London W12 0NN, UK; ^3^Department of Biomedical Sciences, Faculty of Science & Technology, University of Westminster, London W1W 6UW, UK

## Abstract

The long-term effects of antenatal dexamethasone treatment on brain remodelling in 3-month-old male Sprague Dawley rats whose mothers had been treated with dexamethasone were investigated in the present study. Dorsal hippocampus, basolateral amygdala and nucleus accumbens volume, cell numbers, and GFAP-immunoreactive astroglial cell morphology were analysed using stereology. Total brain volume as assessed by micro-CT was not affected by the treatment. The relative volume of the dorsal hippocampus (% of total brain volume) showed a moderate, by 8%, but significant reduction in dexamethasone-treated versus control animals. Dexamethasone had no effect on the total and GFAP-positive cell numbers in the hippocampal subregions, basolateral amygdala, and nucleus accumbens. Morphological analysis indicated that numbers of astroglial primary processes were not affected in any of the hippocampal subregions analysed but significant reductions in the total primary process length were observed in CA1 by 32%, CA3 by 50%, and DG by 25%. Mean primary process length values were also significantly decreased in CA1 by 25%, CA3 by 45%, and DG by 25%. No significant astroglial morphological changes were found in basolateral amygdala and nucleus accumbens. We propose that the dexamethasone-dependent impoverishment of hippocampal astroglial morphology is the case of maladaptive glial plasticity induced prenatally.

## 1. Introduction

Astroglia have been acknowledged to play a role in brain responses to stress and glucocorticoids as its chemical mediators [1, 2]. Such effects implicate brain plasticity and can lead to regional brain remodelling, with volume changes observed within the limbic system in cases of long-term “toxic” stress, and depression and depression-like conditions in humans and animals, respectively [1, 3]. Thus increased amygdala volumes have been observed in teenage adoptees who experienced early life deprivation [4]. On the other hand, hippocampal volume reductions have been reported in patients with PTSD and major depressive disorder with history of early life deprivation [5]. Stress-related reductions in hippocampal volume have also been observed in experimental animals, both rodents [6, 7] and nonhuman primates [8]. Hippocampal volume losses, albeit usually of modest degree, indicate changes in brain tissue architecture, with most studies reporting on neuroplastic rearrangements [9]. However, there is also evidence of astroglial involvement in hippocampal remodelling observed in the rat model of early life deprivation [7].

Also prenatal stress can result in a reduced hippocampal volume associated with suppressed neurogenesis in rhesus monkeys, a phenomenon found to be mediated by corticosteroids [10]. Antenatal treatment with synthetic steroids such as dexamethasone, which cross the placenta [11], is often used in pregnant women at risk for preterm birth [12]. It can, however, affect neurobehavioural development of children who have lower IQ scores and poor motor and visual coordination skills during their school age [13]. About 85% of neonates with antenatal corticosteroid therapy receive multiple courses [14], and dexamethasone is commonly administered to ventilator-dependent premature infants with chronic lung insufficiency to improve lung function [15, 16].

The first human study on postmortem hippocampi of neonates who had been antenatally treated with dexamethasone or betamethasone has shown a glucocorticoid-related reduced density of neurons; no differences have been found in gliosis or myelination [17]. Experimental studies on animals confirm negative effects of antenatal glucocorticoid treatment (AGT) on hippocampal neurogenesis [18]; however, there are no reports about AGT effects on brain glia. The present study addressed this gap in knowledge and hypothesised that rat hippocampal astroglia respond to AGT in a maladaptive manner, in the long term.

## 2. Experimental Procedures

### 2.1. Animals

All animal procedures were carried out in accordance with the United Kingdom Animals Scientific Procedures Act of 1986, at Imperial College London. Sprague Dawley rats (Harlan Olac, Blackthorn, Bicester, Oxfordshire, UK) were kept under controlled lighting (on 0800–2000 h), temperature (21–23°C), and humidity (63%), with standard rat chow and drinking water (except as described below) provided* ad libitum*. Male and female rats were caged separately and allowed to acclimatize to their new environment for one week, after which groups of one male and two female rats were housed together overnight and the presence of vaginal plugs the following morning was taken to confirm mating; pregnancy was confirmed approximately 6 days later by palpation. The timed pregnant rats were housed five per cage until gestational day (GD) 15, when they were caged singly in preparation for giving birth. From GD 19/20 pregnant rats were monitored several times a day and the day of birth was defined as day 0. No more than two progenies per litter (one male, one female) were included in each experimental group in order to minimize potential effects of litter-of-origin. Offspring were weaned at three weeks; male and female progeny were housed separately and they were allowed to grow to adulthood with no further interventions other than normal husbandry. At 68 ± 2 days of age animals were decapitated between 0900 and 1000 h. Male rats were used in the present study.

### 2.2. Dexamethasone Treatment

Dexamethasone was administered noninvasively as dexamethasone sodium phosphate (Faulding Pharmaceuticals Plc., Royal Leamington Spa, UK) in the drinking water given to pregnant rats at GD 16–19, at the dose of 0.5 *μ*g/mL [19] with an estimated daily intake of approximately 75 *μ*g/kg [20]. Withdrawing dexamethasone at GD 19 allowed for clearance of the steroid from the maternal circulation prior to birth, with no observable effects on the outcomes of pregnancy, maternal behaviour, or adult body weight of the offspring [19–21].

### 2.3. pQCT Scanning

Total frozen brain volume (*n* = 6 per group) was analysed by means of peripheral quantitative computed tomography (pQCT) on a Stratec Research SA+ scanner (Stratec Medizintechnik, Pforzheim, Germany) as described elsewhere [7]. Serial coronal CT scans were performed covering entire brain region. Further analysis to measure total brain volume was carried out by using software Avizo (version 5; Mercury Computer Systems, Chelmsford, MA, USA).

### 2.4. Brain Tissue Preparation

Frozen coronal sections (25 *μ*m) were cut in an anterior-posterior direction. The sectioning procedure was designed to yield the following regions of interest (ROIs) (in brackets* A/P* coordinates from bregma, in mm): nucleus accumbens core (3.00 to 2.16), basolateral amygdala (−1.72 to −2.28) and dorsal hippocampus (−2.04 to −4.68) according to the rat brain atlas by Paxinos and Watson [22].

### 2.5. Total Cell Count

Brain sections were stained with hematoxylin (HX) for total cell count and then gradually dehydrated, air-dried, and protected with a histological mounting medium and covered with cover slips.

### 2.6. Immunohistochemistry

To visualise astrocytes, glial fibrillary acidic protein (GFAP) immunohistochemistry was performed [23]. Briefly, peroxidase inactivation was carried out in PBS with 20% methanol, 1.5% H_2_O_2_, and 0.3% Triton X-100; blocking medium contained 0.1% Triton X-100 and 10% normal horse serum, and the anti-GFAP primary antibody (Sigma, UK) was diluted 1 : 500 and applied overnight at 4°C. Controls (blanks) without the primary antibody were processed in parallel. All sections were incubated with the secondary antibody and streptavidin-biotin-peroxidase complex according to manufacturer's instruction (ABC kit, Vector Laboratories, UK). Sections were treated with 3,3′-diaminobenzidine (5 mg/mL, 0.01% H_2_O_2_), rinsed, dehydrated, and mounted in xylene-based Histomount (BDH/Merck, Poole, UK) prior to stereology.

### 2.7. Stereological Procedures

#### 2.7.1. Volume Analysis

**Figure 1:**
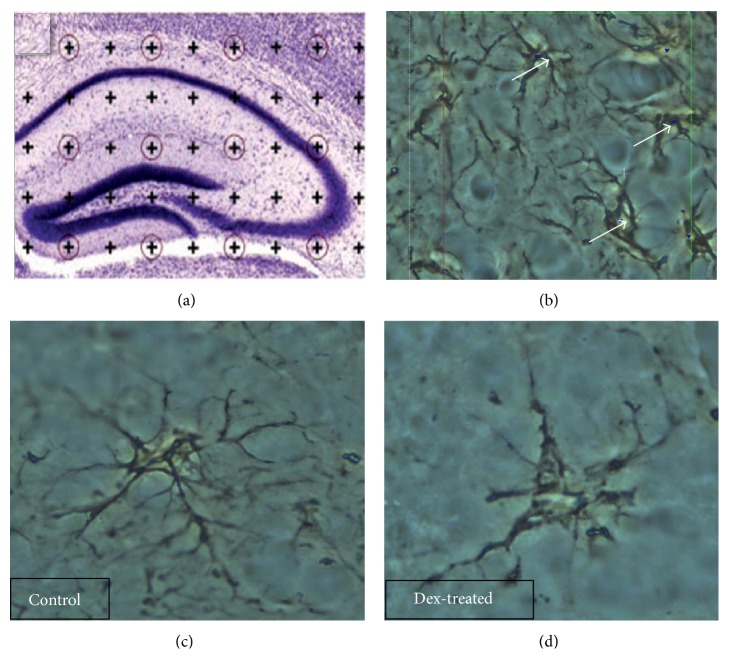
Dorsal hippocampal volume, HX stain (a) and GFAP-positive astroglial cell count (b) estimated by means of stereology. Changes in astroglial morphology in response to AGT: GFAP-positive astroglial cells in control (c) and Dex-treated (d) male rats (dorsal hippocampal dentate gyrus area). GFAP-positive primary process length is reduced in Dex-treated male rats when compared with control, (d) versus (c). Astroglial processes observed at 100x magnification.

Volume estimation using the Cavalieri principle was performed with Stereo-Investigator software (Version 9, MicroBrightField, Inc., Germany) and Olympus BX51 microscope fitted with a motorized stage and video camera on a Zeiss UPlan FLN with a 4x objective (NA = 0.25). Starting at a random position, every tenth section was analyzed, leading to an average of 12 sections for dorsal hippocampus, 6 sections for basolateral amygdala, and 5 sections for nucleus accumbens, per brain. These sets of consecutive sections were used for the estimation of volumes for both control and test groups. The investigator was blind to the experimental groups. A point counting grid (PCG) (i.e., *d* = 50 *μ*m) was used for volume estimation to obtain maximum efficiency. Representative area per point (*a*/*p*) was 2500 *μ*m^2^. After applying the PCG on the sampled sections in a systematic random manner, the number of points hitting region of interest was counted (Figure 1(a)). The efficiency of sampling and volume estimation were checked by estimation of coefficient of error and coefficient of variation [24].

#### 2.7.2. Cell Counts

Cells in the dorsal hippocampal subregions (CA1, CA2, CA3, and DG), basolateral amygdala, and nucleus accumbens were counted using the optical fractionator (Stereo-Investigator, Version 9, MicroBrightField Inc., Germany). An unbiased estimation of total number of cells was achieved by choosing every 10th section according to the systematic random sampling procedure [24] yielding 8 sections per animal. A pilot study was undertaken to determine the following parameters. A sampling area of 160/2500 *μ*m^2^ was found to be optimal for this study. Dissector height was 8 *μ*m and a 1 *μ*m guard zone at the top and bottom part of the section was excluded from the analysis at every step. A fixed counting frame of 50 × 50 and a variable sampling grid size (*x*-*y*-axis) of 150 × 150 CA1, 150 × 150 CA2, 300 × 300 CA3, and 300 × 300 DG were used for the dorsal hippocampus (Figure 1(b)) and 150 × 150 was used for both basolateral amygdala and nucleus accumbens. These sampling grids resulted in 100–200 counting sites per brain. HX and GFAP-positive cells were counted using a 100x Nicon UPlan FLN objective (NA = 1.30) which allowed accurate recognition. Each cell was counted according to the unbiased counting rules. The efficiency of convenient number of sampled cells and parameters was checked by estimation of coefficient of error (CE) and coefficient of variation (CV) [24].

#### 2.7.3. Astroglial Morphology

Morphological analysis was performed on 80 astrocytes in each brain (10 astrocytes per section) using Neurolucida software (Version 9, MicroBrightField, Inc., Germany) for tracing boundaries of the ROIs and astrocytes. Astrocytes within the ROI were traced in a systematic random manner, taking care to avoid those which were superimposed upon other astrocytes or blood vessels. The traced astrocytes were then analysed using Neurolucida explorer software (Version 9, MicroBrightField, Inc., Germany). Morphological analysis was performed by observer who was blind to the experimental groups. Primary process length was measured using 100x objective (Nicon UPlan FLN, NA = 1.30) and by counting the primary processes extending directly from the soma in both the lateral and central quadrants of astrocytes in the same sections.

### 2.8. Statistical Analysis

Using SPSS 17.0 statistical software (SPSS, Inc., USA), ANOVA with a post hoc Tukey test was performed, comparing between group differences. Independent *t*-test was performed to measure difference between two groups. Statistical significance was set at *P* < 0.05. The results are expressed as mean ± SEM.

## 3. Results

### 3.1. Whole Brain Volume and Brain Weight

No significant effects of AGT were observed in the whole brain volume (*P* = 0.376) and brain weight (*P* = 0.442) (Table 1).

### 3.2. Volume Analyses

AGT rats showed a significant decrease in the absolute volume of dorsal hippocampus by 6.5% versus control (*P* < 0.05). When expressed as percentage of the whole brain volume, the volume reduction was 8% (*P* < 0.05) versus control. No significant differences were observed between the treatment groups in the basolateral amygdala (*P* = 0.834) and nucleus accumbens (*P* = 0.905) (Table 2).

### 3.3. HX Stained Total Cell Numbers

AGT did not affect the total cell count (HX stain) in the dorsal hippocampal subregions CA1 (*P* = 0.821), CA2 (*P* = 0.975), CA3 (*P* = 0.991), and DG (*P* = 0.959), basolateral amygdala (*P* = 0.180), and the nucleus accumbens (*P* = 0.474) (Table 3).

### 3.4. GFAP-Positive Astroglial Cell Numbers

There were no significant AGT effects on GFAP-positive cell numbers in the dorsal hippocampal subregions CA1 (*P* = 0.607), CA2 (*P* = 0.517), CA3 (*P* = 0.500), and DG (*P* = 0.424), basolateral amygdala (*P* = 0.495), and the nucleus accumbens (*P* = 0.384) (Table 4).

### 3.5. Astroglia (GFAP-Positive) Cell Morphology

AGT did not affect the numbers of primary processes in the region analysed (Tables 5 and 6) and it had no significant effects on the astroglial morphology in the nonhippocampal regions (Table 5, all group differences at *P* < 0.05).

**Figure 2:**
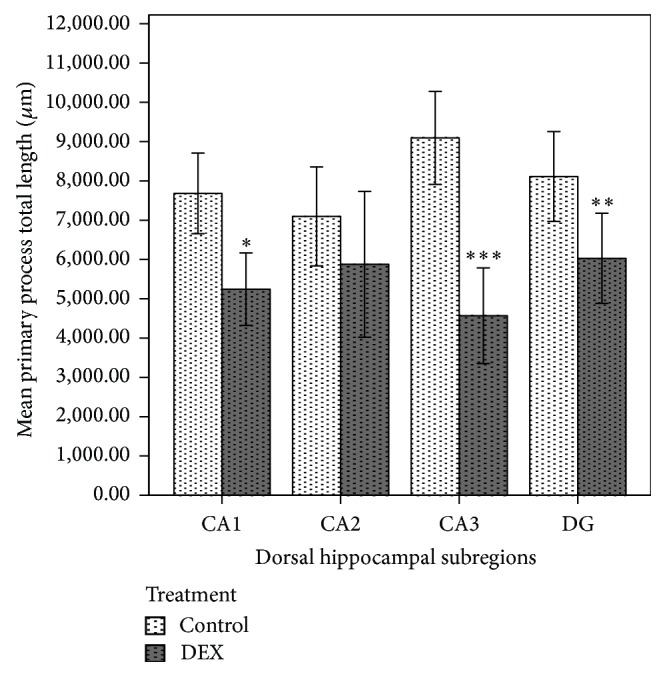
Effect of prenatal dexamethasone treatment on the total length of primary astrocytic processes in hippocampal subregions of male adult Sprague Dawley rats. The total primary process length of GFAP-positive cells in control and dexamethasone-treated groups (*n* = 6 per group). Data are expressed as mean ± SEM. ^*^ *P* < 0.05, ^**^ *P* < 0.01, and ^***^ *P* < 0.001 versus control, independent sample *t*-test.

**Figure 3:**
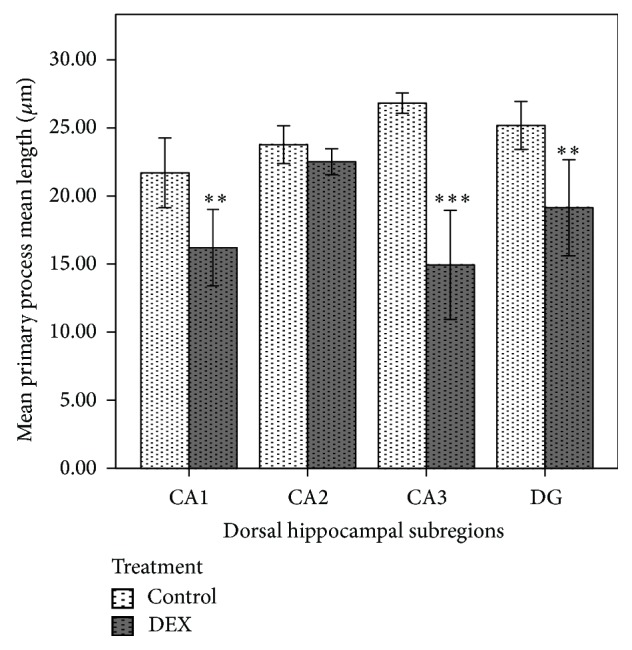
Effect of prenatal dexamethasone treatment on the mean length of primary astrocytic processes in hippocampal subregions of male adult Sprague Dawley rats. The mean primary process length of GFAP-positive cells in control and dexamethasone-treated groups (*n* = 6 per group). Data are expressed as mean ± SEM. ^**^ *P* < 0.01 and ^***^ *P* < 0.001 versus control, independent sample *t*-test.

However, AGT affected the length of primary processes in GFAP-positive cells within the hippocampus (Figures 1(a) and 1(b)); it led to a significant reduction in the total length of astrocytic primary processes in the hippocampal subregions: by 32% in CA1 (*P* < 0.05), 50% in CA3 (*P* < 0.001), and by 25% in DG (*P* < 0.01) (Figure 2). AGT-dependent changes were also observed in the mean length of astrocytic primary processes: reductions by 25% in CA1 (*P* < 0.01), 45% in CA3, (*P* < 0.001), and 25% in DG (*P* < 0.01) (Figure 3).

## 4. Discussion

The present study was focused on the effects of AGT on central astroglia and regional volume of the hippocampus, amygdala, and nucleus accumbens as the areas of the limbic system implicated in response to stress and corticosteroids [2, 3, 9]. AGT was administered to pregnant dams noninvasively, through drinking water, to avoid the stress of animal handling and injections. Of the brain areas tested in adult male rats, the hippocampus responded to AGT, with a long-term reduction in its volume and changes in astroglial morphology. It is plausible that the observed impoverishment of astrocytic primary branches underpins the hippocampal volume reduction as we found no evidence of cell loss. To our knowledge, such AGT-dependent long-term hippocampal remodelling with implications for astroglia has not been reported before.

Astroglia were visualised by means of the commonly used GFAP immunohistochemistry. GFAP is an intermediate filament protein present in cell bodies and primary branches although fine distal processes show little or no GFAP [25]. Although GFAP staining is selective to astrocytes, it does not label all astrocytes in the brain. Accepting these limitations of GFAP immunohistochemistry, the present study focused on the length and numbers of primary astrocytic processes where GFAP is reliably detectable [25]. Stereology was our method of choice as it offers an unbiased approach to regional brain volumetry and morphometry [24].

Previous volumetric studies have considered specific brain regions, expressing their volumes in absolute terms with no reference to the total brain volume, which itself is a variable. Thus the present application of pQCT to measure total brain volume adds the much needed rigour to volumetric assessment where regional volumes need to be scaled to the total brain volume. This is particularly important as perinatal corticosteroid exposure can lead to a decrease in brain weight/volume. For example, a significant brain weight reduction has been reported in rhesus monkeys treated prenatally with corticosteroids [26] and repeated doses of dexamethasone at E17–19 have resulted in a significant brain weight reduction in newborn rats [27]. The present study found no AGT effects on the whole brain weight and volume, the latter measured by pQCT. One of the factors which affect the brain weight and volume is the amount of corticosteroid administered. In the present study a relatively small dose was given which, as discussed previously [19], is in the range used in perinatal medicine and mimics the glucocorticoid activity that is required for normal lung maturation during late gestation.

Within the developing brain, the limbic system, richly expressing glucocorticoid and mineralocorticoid receptors [28], is sensitive to the endogenous and exogenous corticosteroids, which can affect these brain areas with long-term consequences for their structure and function [29]. Reductions in the regional brain volume of the hippocampus and amygdala have been reported in preterm infants [30–32] and a cortical brain volume reduction of 35% has been observed in premature infants treated with dexamethasone [33]. On the other hand, there have been reports of no effect of single betamethasone antenatal dose on regional brain volumes, particularly within the limbic system [34].

In the present study, the hippocampus was significantly affected by AGT, in line with the theory that this region develops primarily during the foetal period [35] although hippocampal volume losses have also been observed following administration of corticosteroids in adult monkeys and rats [36, 37]. No changes were seen in the basolateral amygdala and nucleus accumbens volume, which suggests a region-selective treatment effect consistent with the density of glucocorticoid receptor distribution [28].

A transient reduction in hippocampal volume has been observed in adolescent but not adult mice exposed to a clinically relevant dose of dexamethasone at E15 [18]. The extent of hippocampal volume loss is normally mild/moderate. The present reduction of the hippocampal volume by 8% fits in that range while being statistically significant. It is comparable with some previous reports on stress/steroid-dependent losses in the hippocampal volume across several species. Thus Coe et al. [10] have found hippocampal volume reductions by 12% and 10% in the offspring of rhesus monkeys exposed to early life stress. In tree shrews, four weeks' cortisol treatment or psychosocial stress has resulted in a hippocampal volume loss by 5–10% [38]. Chronic social defeat stress has led to a similarly mild yet significant hippocampal volume loss in adult rats [6]. Human adolescents who were born very preterm have been found to have a hippocampal volume loss in the range of 12% to 15.6% [39].

What causes the hippocampal volume losses in responses to stress/glucocorticoids? There is a range of factors such as neuronal and/or glial cell death, reduction of cell size, including atrophy of neuronal dendrites and/or glial branches, reduced water content or impaired neurogenesis [1], and/or reduced microvasculature [6]. The present study did not find cell losses in the brain areas tested and focused on astroglia because of their major role in maintaining brain plasticity at the level of synapses and cells [25]. Currently, published studies on the mechanisms underlying glucocorticoid-induced brain volume reductions have focused on neurons. As a result, neuronal cell loss has been well researched and documented, and various dexamethasone treatments have been found to lead to neuronal loss in rats [40, 41] and primates [26, 37] mostly in the hippocampal CA3 and dentate gyrus. Less attention has been given to astroglial cells, despite their role in the maintenance of brain homeostasis and plasticity [25].

The impoverishment in astroglial morphology reported in the present study is consistent with the known glia-inhibitory effects of exogenous glucocorticoids or stress* in vitro* [42, 43] and* in vivo* [44]. Treatment with corticosterone or synthetic glucocorticoids such as dexamethasone has been known to inhibit GFAP expression in the neonatal and adult rat brain [45–48] thus suggesting that astrocytes can be affected by glucocorticoid overload. The very fact that dexamethasone can affect GFAP expression raises a valid methodological consideration as to what extent the present observations of changes in the morphology of GFAP-positive astroglia are affected by methodological bias when comparing between the groups. Central astroglia are heterogeneous [25, 49] and using GFAP as the only immunohistochemical marker brings in an intrinsic limitation, shared by many current studies, as critically reviewed elsewhere [49]. However, the present GFAP responses to AGT appear to be selective to the hippocampus, where astroglial primary branches reduced in length but no GFAP-positive cell losses were found. Such a discrete response of GFAP immunoreactivity implies that dexamethasone, administered at the present low dose as AGT, did not induce a global effect in GFAP expression. This not only highlights the unique susceptibility of hippocampal regions but also suggests that the changes observed here are true manifestations of impoverished hippocampal astroglial morphology in response to AGT.

It is increasingly evident that nongenetic/environmental factors acting in early life result in long-term alterations of the physiological systems affected. Perinatal programming and/or plasticity of the physiological system under extreme conditions such as “toxic” stress or AGT can result in long-term neurobehavioural abnormalities [3, 29, 50] and there is growing evidence that prenatal or early postnatal exposure to stressors affects the long-term brain functioning [51].

To conclude, this study reports significant maladaptive changes in astroglia in the hippocampal subregions CA1, CA3 and dentate gyrus, as a long-term effect of AGT. It is plausible to assume that the reductions in astrocytic primary branches contribute to the losses in the hippocampal volume. They are also likely to affect astroglial function and neuron-glia interactions as retraction of primary astroglial processes can disadvantage local networks formed with glia and neurons. The observed dexamethasone-induced impoverishment of astroglial morphology can be treated as a demonstration of glial maladaptive plasticity, a phenomenon that deserves more research in the context of effects of corticosteroid overload and stress-related pathologies. Epigenetics of this phenomenon should be of interest considering the clinical use of dexamethasone.

## Figures and Tables

**Table 1:** Effects of AGT exposure on brain weight and volume of male adult Sprague Dawley rats.

	Control	Dex-treated
Brain weight (g)	1.74 ± 0.02	1.77 ± 0.03
Brain volume (mm^3^)	1650.18 ± 17.75	1681.24 ± 28.43

The brain weight and volume are expressed as mean ± SEM (*n* = 6 per group). No significant differences between Dex-treated (AGT) and control were observed.

**Table 2:** Effects of AGT on the regional brain volume of male adult Sprague Dawley rats.

Volume		
	Control	Dex-treated
Absolute values (mm^3^)		
Dorsal hippocampus	32.8 ± 0.46	30.8 ± 0.50^*^
Basolateral amygdala	0.81 ± 0.02	0.80 ± 0.02
Nucleus accumbens	2.28 ± 0.06	2.26 ± 0.07
Relative values (as % brain volume)		
Dorsal hippocampus	1.99 ± 0.04	1.83 ± 0.04^*^
Basolateral amygdala	0.046 ± 0.001	0.048 ± 0.001
Nucleus accumbens	0.138 ± 0.004	0.135 ± 0.005

The volume of the dorsal hippocampus, basolateral amygdala, and nucleus in control and dexamethasone-treated groups (*n* = 6 per group). Data are expressed as mean ± SEM. ^*^ *P* < 0.05 versus control, independent sample *t*-test.

**Table 3:** Effects of AGT on the number of total (hematoxylin-stained) and GFAP-positive cellsin dorsal hippocampus of male adult Sprague Dawley rats.

Subregion	Control	Dex-treated
Hematoxylin stained cell numbers estimated per region (×10^3^)		
CA1	706.8 ± 8.8	714.1 ± 29.7
CA2	95.9 ± 3.4	95.8 ± 2
CA3	491.3 ± 37.2	490.7 ± 32.6
DG	791.4 ± 31.6	789.1 ± 30.6

GFAP +ve cell numbers estimated per region (×10^3^)		
CA1	219.4 ± 6	213.6 ± 9.1
CA2	26.8 ± 1.1	25.8 ± 1
CA3	110.9 ± 6.1	104.4 ± 6.9
DG	201 ± 4.7	195.3 ± 5.1

The numbers of total and GFAP-positive cells are shown for control and dexamethasone-treated groups (*n* = 6 per group). Data are expressed as mean ± SEM. No significant differences between Dex-treated (AGT) and control were observed.

**Table 4:** Effects of AGT on the number of total and GFAP-positive cells in basolateral amygdala and nucleus accumbens region of cells in male adult Sprague Dawley rats.

Subregion	Control	Dex-treated
Hematoxylin stained cell numbers estimated per region (×10^3^)		
Basolateral amygdala	52.86 ± 3.4	58.79 ± 2.4
Nucleus accumbens	146.58 ± 6.8	139.61 ± 6.4

GFAP +ve cell numbers estimated per region (×10^3^)		
Basolateral amygdala	23.68 ± 1.9	25.71 ± 2.1
Nucleus accumbens	20.73 ± 1.8	18.28 ± 2.0

The numbers of total and GFAP-positive cells in control and dexamethasone-treated groups (*n* = 6 per group). Data are expressed as mean ± SEM. No significant differences between Dex-treated (AGT) and control were observed.

**Table 5:** Effects of AGT on the morphology of GFAP-positive astroglia in the basolateral amygdala and nucleus accumbens region of male adult Sprague Dawley rats.

GFAP-astroglia morphology		
	Control	Dex-treated
Total primary process length (*µ*m)		
Basolateral amygdala	5761.8 ± 582.6	6123.5 ± 598.9
Nucleus accumbens	4033.4 ± 403.9	3890.1 ± 489.2
Primary process mean length		
Basolateral amygdala	26.8 ± 1.6	27.5 ± 2.1
Nucleus accumbens	22.7 ± 1.8	20.8 ± 1.7
Number of primary processes		
Basolateral amygdala	213 ± 11.4	221 ± 9.1
Nucleus accumbens	178 ± 8.5	185 ± 10.6

The total primary process length, mean primary process length, and numbers of primary processes of GFAP-positive cells in control and dexamethasone-treated groups (*n* = 6 per group). Data are expressed as mean ± SEM. No significant differences between Dex-treated (AGT) and control were observed.

**Table 6:** Effects of AGT on GFAP-positive astroglial primary process numbers in male adult Sprague Dawley rats.

GFAP-astroglia morphology		
	Control	Dex-treated
Number of primary process		
CA1	362 ± 36.1	323.3 ± 4.4
CA2	301 ± 26.2	311 ± 26.4
CA3	339 ± 16.9	305.7 ± 8.1
DG	321.7 ± 14.3	314.8 ± 4.7

The numbers of primary processes of GFAP-positive cells in control and dexamethasone-treated groups (*n* = 6 per group). Data are expressed as mean ± SEM. No significant differences between Dex-treated (AGT) and control were observed.

## Abbreviations

AGT: Antenatal glucocorticoid treatment
Dex: Dexamethasone
GD: Gestational day
GFAP: Glial fibrillary acidic protein
HX: Hematoxylin
MDD: Major depressive disorder
PBS: Phosphate-buffered saline
PTSD: Posttraumatic stress disorder
ROI: Region of interest.

## References

[1] Czéh B., Lucassen P. J. (2007). What causes the hippocampal volume decrease in depression? Are neurogenesis, glial changes and apoptosis implicated?. *European Archives of Psychiatry and Clinical Neuroscience*.

[2] Lucassen P. J., Pruessner J., Sousa N. (2014). Neuropathology of stress. *Acta Neuropathologica*.

[3] McEwen B. S., Eiland L., Hunter R. G., Miller M. M. (2012). Stress and anxiety: structural plasticity and epigenetic regulation as a consequence of stress. *Neuropharmacology*.

[4] Mehta M. A., Golembo N. I., Nosarti C. (2009). Amygdala, hippocampal and corpus callosum size following severe early institutional deprivation: the English and Romanian Adoptees study pilot.. *Journal of Child Psychology and Psychiatry, and Allied Disciplines*.

[5] Frodl T., O'Keane V. (2013). How does the brain deal with cumulative stress? A review with focus on developmental stress, HPA axis function and hippocampal structure in humans. *Neurobiology of Disease*.

[6] Czéh B., Abumaria N., Rygula R., Fuchs E. (2010). Quantitative changes in hippocampal microvasculature of chronically stressed rats: no effect of fluoxetine treatment. *Hippocampus*.

[7] Shende V. H., Pryce C. R., Rüedi-Bettschen D., Rae T. C., Opacka-Juffry J. (2012). Evidence for hippocampal remodeling as a long term effect of early life stress in the rat model of early deprivation. *Recent Developments in Brain Research*.

[8] Jackowski A., Perera T. D., Abdallah C. G. (2011). Early-life stress, corpus callosum development, hippocampal volumetrics, and anxious behavior in male nonhuman primates. *Psychiatry Research—Neuroimaging*.

[9] Pittenger C., Duman R. S. (2008). Stress, depression, and neuroplasticity: a convergence of mechanisms. *Neuropsychopharmacology*.

[10] Coe C. L., Kramer M., Czéh B. (2003). Prenatal stress diminishes neurogenesis in the dentate gyrus of juvenile Rhesus monkeys. *Biological Psychiatry*.

[11] Funkhouser J. D., Peevey K. J., Mockridge P. B., Hughes E. R. (1978). Distribution of dexamethasone between mother and fetus after maternal administration. *Pediatric Research*.

[12] Roberts D., Dalziel S. (2006). Antenatal corticosteroids for accelerating fetal lung maturation for women at risk of preterm birth. *Cochrane Database of Systematic Reviews*.

[13] Yeh T. F., Lin Y. J., Lin H. C. (2004). Outcomes at school age after postnatal dexamethasone therapy for lung disease of prematurity. *The New England Journal of Medicine*.

[14] Empana J. P., Anceschi M. M., Szabo I., Cosmi E. V., Breart G., Truffert P. (2004). Antenatal corticosteroids policies in 14 European countries: factors associated with multiple courses. The EURAIL survey. *Acta Paediatrica, International Journal of Paediatrics*.

[15] Romagnoli C., Zecca E., Luciano R., Torrioli G., Tortorolo G. (2002). A three year follow up of preterm infants after moderately early treatment with dexamethasone. *Archives of Disease in Childhood: Fetal and Neonatal Edition*.

[16] Charil A., Laplante D. P., Vaillancourt C., King S. (2010). Prenatal stress and brain development. *Brain Research Reviews*.

[17] Tijsseling D., Wijnberger L. D. E., Derks J. B. (2012). Effects of antenatal glucocorticoid therapy on hippocampal histology of preterm infants. *PLoS ONE*.

[18] Noorlander C. W., Tijsseling D., Hessel E. V. S. (2014). Antenatal glucocorticoid treatment affects hippocampal development in mice. *PLoS ONE*.

[19] McArthur S., McHale E., Gillies G. E. (2007). The size and distribution of midbrain dopaminergic populations are permanently altered by perinatal glucocorticoid exposure in a sex- region- and time-specific manner. *Neuropsychopharmacology*.

[20] McArthur S., McHale E., Dalley J. W., Buckingham J. C., Gillies G. E. (2005). Altered mesencephalic dopaminergic populations in adulthood as a consequence of brief perinatal glucocorticoid exposure. *Journal of Neuroendocrinology*.

[21] Theogaraj E., John C. D., Christian H. C., Morris J. F., Smith S. F., Buckingham J. C. (2005). Perinatal glucocorticoid treatment produces molecular, functional, and morphological changes in the anterior pituitary gland of the adult male rat. *Endocrinology*.

[22] Paxinos G., Watson C. (2005). *The Rat Brain in Stereotaxic Coordinates*.

[23] Leventopoulos M., Rüedi-Bettschen D., Knuesel I., Feldon J., Pryce C. R., Opacka-Juffry J. (2007). Long-term effects of early life deprivation on brain glia in Fischer rats. *Brain Research*.

[24] West M. J., Slomianka L., Gundersen H. J. G. (1991). Unbiased stereological estimation of the total number of neurons in the subdivisions of the rat hippocampus using the optical fractionator. *Anatomical Record*.

[25] Theodosis D. T., Poulain D. A., Oliet S. H. R. (2008). Activity-dependent structural and functional plasticity of astrocyte-neuron interactions. *Physiological Reviews*.

[26] Uno H., Lohmiller L., Thieme C. (1990). Brain damage induced by prenatal exposure to dexamethasone in fetal rhesus macaques. I. Hippocampus. *Developmental Brain Research*.

[27] Carlos R. Q., Seidler F. J., Slotkin T. A. (1992). Fetal dexamethasone exposure alters macromolecular characteristics of rat brain development: a critical period for regionally selective alterations?. *Teratology*.

[28] de Kloet E. R. (2000). Stress in the brain. *European Journal of Pharmacology*.

[29] Matthews S. G. (2001). Antenatal glucocorticoids and the developing brain: mechanisms of action. *Seminars in Neonatology*.

[30] Abernethy L. J., Palaniappan M., Cooke R. W. I. (2002). Quantitative magnetic resonance imaging of the brain in survivors of very low birth weight. *Archives of Disease in Childhood*.

[31] Isaacs E. B., Lucas A., Chong W. K. (2000). Hippocampal volume and everyday memory in children of very low birth weight. *Pediatric Research*.

[32] Peterson B. S., Vohr B., Staib L. H. (2000). Regional brain volume abnormalities and long-term cognitive outcome in preterm infants. *Journal of the American Medical Association*.

[33] Murphy B. P., Inder T. E., Huppi P. S. (2001). Impaired cerebral cortical gray matter growth after treatment with dexamethasone for neonatal chronic lung disease. *Pediatrics*.

[34] Yossuck P., Kraszpulski M., Salm A. K. (2006). Perinatal corticosteroid effect on amygdala and hippocampus volume during brain development in the rat model. *Early Human Development*.

[35] Rice D., Barone S. (2000). Critical periods of vulnerability for the developing nervous system: evidence from humans and animal models. *Environmental Health Perspectives*.

[36] Woolley C. S., Gould E., McEwen B. S. (1990). Exposure to excess glucocorticoids alters dendritic morphology of adult hippocampal pyramidal neurons. *Brain Research*.

[37] Sapolsky R. M., Uno H., Rebert C. S., Finch C. E. (1990). Hippocampal damage associated with prolonged glucocorticoid exposure in primates. *The Journal of Neuroscience*.

[38] Ohl F., Michaelis T., Vollmann-Honsdorf G. K., Kirschbaum C., Fuchs E. (2000). Effect of chronic psychosocial stress and long-term cortisol treatment on hippocampus-mediated memory and hippocampal volume: a pilot-study in tree shrews. *Psychoneuroendocrinology*.

[39] Nosarti C., Al-Asady M. H. S., Frangou S., Stewart A. L., Rifkin L., Murray R. M. (2002). Adolescents who were born very preterm have decreased brain volumes. *Brain*.

[40] Haynes L. E., Griffiths M. R., Hyde R. E., Barber D. J., Mitchell I. J. (2001). Dexamethasone induces limited apoptosis and extensive sublethal damage to specific subregions of the striatum and hippocampus: implications for mood disorders. *Neuroscience*.

[41] Sousa N., Paula-Barbosa M. M., Almeida O. F. X. (1999). Ligand and subfield specificity of corticoid-induced neuronal loss in the rat hippocampal formation. *Neuroscience*.

[42] Crossin K. L., Tai M.-H., Krushel L. A., Mauro V. P., Edelman G. M. (1997). Glucocorticoid receptor pathways are involved in the inhibition of astrocyte proliferation. *Proceedings of the National Academy of Sciences of the United States of America*.

[43] Sabolek M., Herborg A., Schwarz J., Storch A. (2006). Dexamethasone blocks astroglial differentiation from neural precursor cells. *NeuroReport*.

[44] Nichols N. R., Masters J. N., Finch C. E. (1990). Changes in gene expression in hippocampus in response to glucocorticoids and stress. *Brain Research Bulletin*.

[45] Nichols N. R., Osterburg H. H., Masters J. N., Millar S. L., Finch C. E. (1990). Messenger RNA for glial fibrillary acidic protein is decreased in rat brain following acute and chronic corticosterone treatment. *Molecular Brain Research*.

[46] Laping N. J., Nichols N. R., Day J. R., Finch C. E. (1991). Corticosterone differentially regulates the bilateral response of astrocyte mRNAs in the hippocampus to entorhinal cortex lesions in male rats. *Molecular Brain Research*.

[47] Tsuneishi S., Takada S., Motoike T., Ohashi T., Sano K., Nakamura H. (1991). Effects of dexamethasone on the expression of myelin basic protein, proteolipid protein, and glial fibrillary acidic protein genes in developing rat brain. *Developmental Brain Research*.

[48] O'Callaghan J. P., Brinton R. E., McEwen B. S. (1991). Glucocorticoids regulate the synthesis of glial fibrillary acidic protein in intact and adrenalectomized rats but do not affect its expression following brain injury. *Journal of Neurochemistry*.

[49] Sofroniew M. V., Vinters H. V. (2010). Astrocytes: biology and pathology. *Acta Neuropathologica*.

[50] Maccari S., Darnaudery M., Morley-Fletcher S., Zuena A. R., Cinque C., van Reeth O. (2003). Prenatal stress and long-term consequences: implications of glucocorticoid hormones. *Neuroscience and Biobehavioral Reviews*.

[51] Pryce C. R., Aubert Y., Maier C., Pearce P. C., Fuchs E. (2011). The developmental impact of prenatal stress, prenatal dexamethasone and postnatal social stress on physiology, behaviour and neuroanatomy of primate offspring: Studies in rhesus macaque and common marmoset. *Psychopharmacology*.

